# Endocrine disorders in Rett syndrome: a systematic review of the literature

**DOI:** 10.3389/fendo.2024.1477227

**Published:** 2024-10-31

**Authors:** Giorgia Pepe, Roberto Coco, Domenico Corica, Giovanni Luppino, Letteria Anna Morabito, Cecilia Lugarà, Tiziana Abbate, Giuseppina Zirilli, Tommaso Aversa, Stefano Stagi, Malgorzata Wasniewska

**Affiliations:** ^1^ Unit of Pediatrics, Department of Human Pathology of Adulthood and Childhood, University of Messina, Messina, Italy; ^2^ Department of Health Sciences, University of Florence, Florence, Italy

**Keywords:** Rett syndrome, endocrinopathy, MECP2 deletion, CDKL5 deletion, epilepsy

## Abstract

**Background:**

Rett syndrome (RTT) is an X-linked progressive neurodevelopmental disorder that involves mainly girls and is the second most frequent cause of genetic intellectual disability. RTT leads to neurological regression between 6 and 18 months of life and could be associated with a variable neurological impairment. However, RTT affects not only neurological function but also wide aspects of non-neurological organs. Recent data showed that the endocrine system is often involved in RTT patients, including disorders of growth, bone health, thyroid, puberty onset, and weight abnormalities However, systematic data on endocrinopathies in RTT are scarce and limited.

**Objective:**

This review aims to analyze the prevalence and type of endocrine comorbidities in RTT population, to allow a precocious diagnosis and appropriate endocrinological management.

**Methods:**

Systematic research was carried out from January 2000 to March 2024 through MEDLINE via PubMed, Scopus, and the Cochrane Library.

**Results:**

After the selection phase, a total of 22 studies (1090 screened) met the inclusion criteria and were reported in the present review. Five studies were observational-retrospective, four were cross-sectional and case report or series, three were survey, prospective, and case-control, and finally one study for descriptive-transversal and longitudinal population-based study. The sample population consisted of multiethnic groups or single ethnic groups. The main endocrinopathies reported were malnutrition, bone alterations, and alterations of puberty onset.

**Conclusions:**

Our analysis shows that endocrinopathies are not rare in RTT patients. Therefore, in the context of a multidisciplinary approach, accurate screening and monitoring for endocrinopathies should be recommended in all RTT patients, to improve clinical practice, healthcare management, and, finally, patients’ quality of life.

## 1 Introduction

Rett syndrome (RTT; OMIM ID 312750) is a severe neurodevelopmental disorder that has been identified almost exclusively in females, mainly after 6 months of age (1). It affects about 1 in 15,000 newborns (2). It is the second genetic cause of intellectual disability in girls after Down syndrome (3). In 90–95% of the cases, mutations in Methyl-CpG binding protein 2 gene are responsible for most typical RTT and a smaller proportion of atypical RTT. On the other hand, patients with Rett phenotype together with early-onset epilepsy caused by mutations in the cyclin-dependent kinase-like 5 gene (*CDKL5*) (4). Another gene known as *FOXG1* has been associated with atypical RTT or a RTT-like phenotype, and may manifest with preserved function and specific clinical features (5). In 1999 the first mutations in the methyl-CpG binding protein-2 (*MeCP2*) gene were described. The *MeCP2*gene codes for the methyl-CpG binding protein-2 (*MeCP2*) which is involved in the long-term silencing of genes and is expressed in all tissues (6). Mutations in the *MeCP2*gene, primarily causing a loss of function, are mainly responsible for RTT, a disorder affecting the X chromosome (7). Since roughly 95% of the mutations occur (*de novo*), prenatal testing and/or genetic counseling for Rett syndrome is often not helpful. *MeCP2* plays a pivotal role in brain functioning and neuronal development, both at the beginning of neuronal differentiation and thereafter (8).

RTT patients begin life apparently ‘healthy’. However, from 6 to 18 months of age, they undergo regression of early milestones, with deterioration of motor skills, eye contact, speech, and motor control, deceleration of head growth, and development of distinctive repetitive, purposeless hand movements (9). A spectrum of neurological issues, including anxiety, breathing problems (respiratory dysrhythmias), and seizures, usually develop over time (10). The clinical phenotype of RTT is highly variable and can be classified into two main categories: typical (classic) RTT and atypical (variant) RTT. Diagnostic criteria for typical RTT require a period of regression, followed by recovery or stabilization, and fulfillment of all the main criteria (loss of purposeful hand skills, loss of spoken language, gait abnormalities, and stereotypic hand movements) (3). Further manifestations can include autistic features, intermittent breathing abnormalities, autonomic nervous system dysfunction, cardiac abnormalities, and sleep disturbances. In addition to typical or classical RTT, some individuals may present with many, but not all the clinical features of RTT, thus there are ‘variant’ or ‘atypical’ RTT (11). These include three main variants: preserved speech, early onset seizure, and congenital variants (12). Trofinetide is at the moment the only disease-modifying therapy for RTT approved by FDA since 2023, and it is a potential effective and safe therapeutic opportunity (13). Different pharmacologic drugs, including glatiramer acetate and dextromethorphan have been investigated in small clinical trials but with modest benefits (14). Gene therapy, which is nowadays in the drug development phase, may promise new cure opportunities (15).

Initially, RTT was considered a purely nervous system pathology, but in recent years it has emerged as a complex and heterogeneous multisystemic disease, with a variety of clinical appearances (16). Although neurological conditions are predominant, the disease affects not only the central nervous system but also a wide array of non-neurological organs. Recent studies showed that multisystemic comorbidities, like gastrointestinal, orthopedic, endocrine, or cardiac issues, may be more or less prevalent in RTT patients (17). Concerning endocrine disorders in RTT, data are few and contrasting. Even if endocrinopathies are less common among comorbidities, they seem to be significantly more frequent than in the general population. Endocrine disorders have a considerable impact mainly on growth, weight, menstrual cycles, and bones. Some authors reported low bone mineral content as the most common endocrine disorder in RTT (18), followed by alterations in the timing of pubertal onset and menarche (19). Furthermore, thyroid function is a matter of great concern in these patients, considering the effect of thyroid hormones (TH) on proper mammalian brain development (20).

Overall, data on endocrinopathies in RTT patients is still scarce and univocal. This systematic review aims to describe the prevalence and type of the main endocrine comorbidities, focusing especially but not exclusively on pediatric age, providing a proposal for endocrinological management of RTT patients.

## 2 Methods

We performed the review following the PRISMA 2020 guidelines (21). Systematic research was performed, covering the last 24 years (from January 2000 to March 2024), according to the PRISMA statement, through MEDLINE via PubMed, the Cochrane Library, and Scopus databases, to find studies reporting endocrinopathies in patients with genetic diagnosis of Rett syndrome. The research was based on the combination of “Rett syndrome” with all the following keywords: “thyroid”, “growth”, “short stature”, “obesity”, “malnutrition”, “puberty”, “menstruation”, “menstrual irregularities”, “hyperprolactinemia” and “bone” to include a variety of results. No specific registers of RTT population were used for the systematic research. Literature before 2000 was not included because the main causative genetic mutation in RTT was identified in 1999 (7, 22).

The settled inclusion criteria were: articles written in English, belonging to the categories of a review, clinical study, clinical trial, clinical trial protocol, multicenter study, randomized controlled trial, and observational study, which report endocrinopathies in genetically confirmed RTT in pediatric or mixed population (both adolescents and adults patients). Due to the rarity of studies in this field, case reports and small cohorts were also included. The exclusion criteria were letters to editors, articles belonging to only the adult population, not full-text articles, absence of genetic diagnosis of RTT and experimental studies (for example murine studies). From our initial research, all the studies reporting the presence of one or more endocrinopathies in RTT patients were subsequently reviewed individually, hence, we focused on: the type of endocrinopathy, incidence and/or prevalence, specific gene mutation, presence or absence of epilepsy, and eventually anticonvulsant drugs.

Titles and abstracts of all retrieved articles were screened by four authors (R.C., G.L., C.L., T.A.) to identify articles for full-text review. All authors assessed the eligibility of all full-text articles.

### 2.1 Data items

The following information was extracted from the included studies: bibliographic data (first author, publication year, country), study characteristics (study design), participant characteristics (sample size, gender, age at onset, Rett mutation, epilepsy), outcome (prevalence of endocrinopathies). Equations

## 3 Results

**Figure 1:**
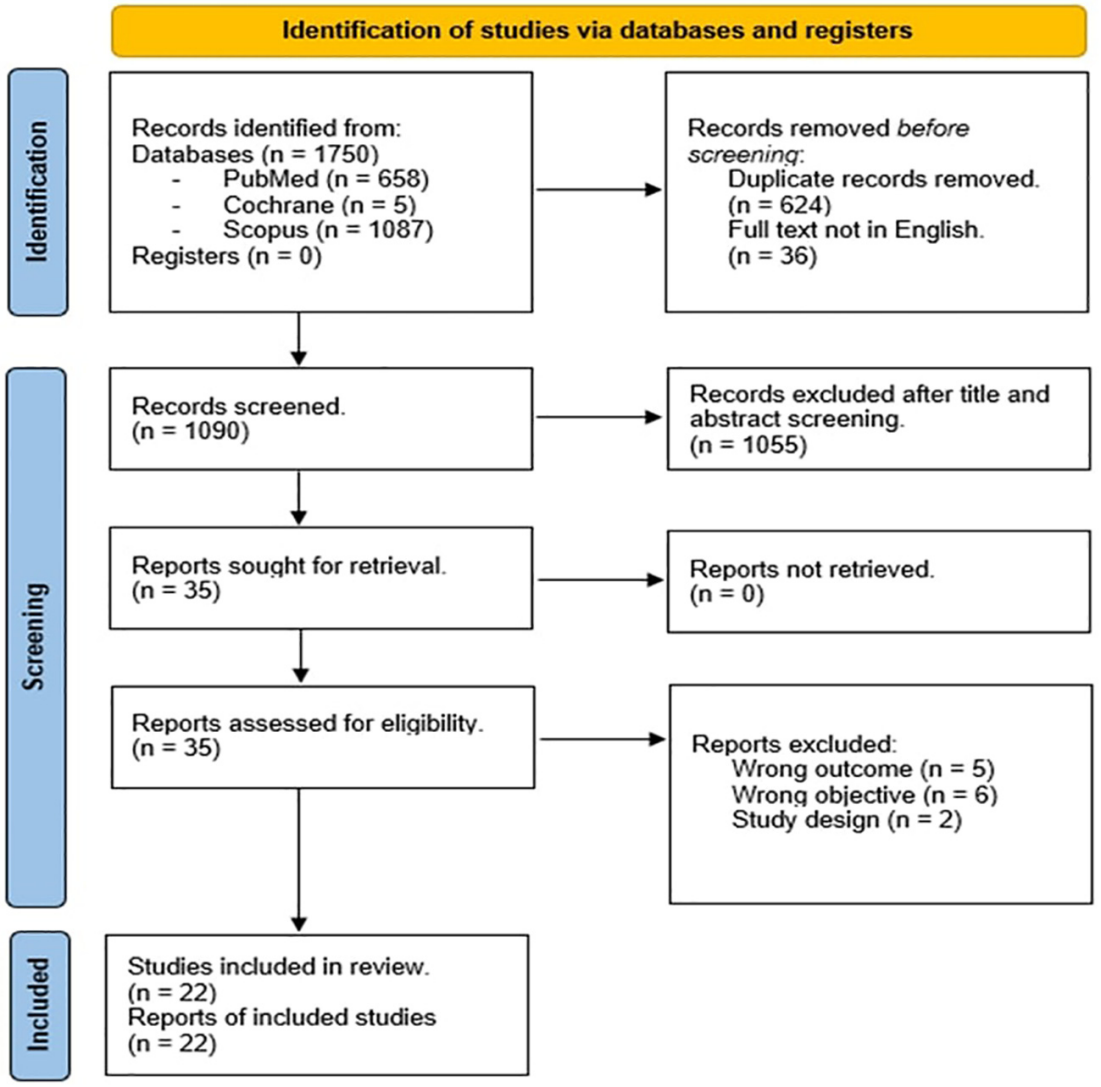
PRISMA 2020 flow diagram of the study.

We identified 1750 records. After duplicates and not in English full-text removal, we screened 1090 records, from which were viewed 1090 full-text studies, and finally included 22 articles (19, 23–43) after title, abstract, and PICO evaluation (**Figure 1**). As above mentioned, we did not carry out meta-analyses since the studies included were not homogeneous in terms of the type of study design, number of cohorts, age, and race difference among populations. Five studies were observational-retrospective, four were cross-sectional, three were case reports or series, three were survey, three were prospective, two comprehended case-control, and finally one study for descriptive-transversal and longitudinal population-based study. The sample population consisted of multiethnic groups or single ethnic groups. Nine studies (40.90%) were from the USA, seven (31.82%) from Europe, three (13.64%) from Australia and the remaining three studies (13.64%) were from Taiwan, Japan, and Brazil. Thirteen (59.10%) studies were published after 2012. Only two studies included male patients together with a female population. Seven studies included exclusively RTT patients < 18 years old while the other articles involved both adults and adolescents. Interestingly only one study included FOX1 gene mutation together with *MeCP2* and *CDKL5* mutations, and two studies reported patients with *CDKL5* mutation and *MeCP2*. The main endocrinopathies described in the selected studies were malnutrition and bone alterations (8/24) followed by puberty onset disorders and obesity (7/24). Short stature was identified in 6/24 studies, menstrual irregularities in 5/24, thyroid disorders were presented in 2/24 studies, and hyperprolactinemia in only 1/24 study. Finally, seizure disorders were described as RTT comorbidities in fourteen studies (63.60%). The main characteristics of the included studies are reported in **Table 1**.

**Table 1:** The Table summarizes the main data of the studies reporting endocrine disorders in Rett syndrome patients.

PAPER, YEAR, COUNTRY	DESIGN	N. PATIENTS	AGE OF EVALUATION	SEX/RACE	GENETIC	ENDOCRINOPATHIES	EPILEPSY
**Pepe et al., 2024 (** 23) **Italy**	Retrospective observational study	51	9.65 ±5.9 years	47 *female*, 4 *male* *Caucasian*	*MeCP2* (74.5%), *CDKL5* (21.6%), *FOXG1* (3.9%)	*Short stature* (47.1%), *Menstrual irregularities* (46.2%), *Malnutrition* (25.5%), *Obesity* (19.6%), *Bone alterations* (19.6%), *Precocious puberty* (15.4%), *Hyperprolactinemia* (13.7%), *Thyroid alterations* (9.8%).	*Epilepsy disorders* (72.5%)
**Stagi et al., 2014 (** 24) **Italy**	Case-control Control: 146 age-matched healthy Caucasian girls	45	8.6 ± 5.3 years	45 *female*, 0 *male* *Caucasian*	*MeCP2* (97%), *CDKL5* (13%)	*Thyroid alterations*: - 17.7% showed FT4 levels higher than the upper references limit. - 26.7% showed FT3 levels higher than the upper references limit - 10.2% showed TSH high levels	*Epilepsy disorders* (29%)
**Wong et al., 2021 (** 25) **Taiwan**	Cross-sectional	44	17.03 ± 10.52 years 54.55% <18 years	44 *female*, 0 *male* *Taiwanese*	*MeCP2* (100%)	*Short stature* 41.6% *Malnutrition* 37.5% *Obesity* 4.1%	*N/A*
**Huppke et al., 2001 (** 26) **Germany**	Prospective	38	Median 7.1 years (range 2.1 to 17 years)	38 *female*, 0 *male* *Caucasian (from Germany)*	*N/A*	*Short stature* 91.1%	*N/A*
**Tarquinio et al., 2012 (** 27) **USA**	Observational study From the multicenter RTT Natural History Study (RNHS)	816	345 <10 yo 252 > 10 yo	816 *female*, 0 *male* *Caucasian* 88% *African* *American* 6% *Asian* 5% *American Indian* 1%	*MeCP2* (92%)	Severe somatic growth deficit with microcephaly (29.2%) *Precocious puberty* 12% *Late-onset puberty* 3%	*N/A*
**Reilly et al., 2001 (** 28) **Australia**	Retrospective Observational study	59	2.1-44.9 years old (Mean: 12.6 years SD 11.04 years).	59 *female*, 0 *male* *Caucasian*	*N/A*	*Malnutrition* 26%	*Epilepsy disorders* (62.7%)
**Schwartzman et al., 2008 (** 29) **Brazil**	*Descriptive and transversal study*	27	Between the ages of 2.6 and 21.8 years	27 *female*, 0 *male* *Brasilian*	*N/A*	*Short stature* 48.1% *Malnutrition* 37% *Obesity* 7.4%	*Epilepsy disorders* (69.2%)
**Czerwonogrodzka-Senczyna et al., 2023 (** 30) **Poland**	*Case-control* *Control: 22 healthy female*	49	8.7 ± 4.9 years	49 *female*, 0 *male* *Polish*	*Common MeCP2 mutations 51%*	*Malnutrition* 38.78% *Obesity* 10.2%	*N/A*
**Motil et al., 2012 (** 31) **USA**	*Survey* From the North American RTT database	983	0–5 y: 12%; 6–10 y: 22%; 11–14 y: 12%; 15–19 y: 17%; 20–29 y: 24%; 30+ y: 13%	983 *female*, 0 *male* N/A	*MeCP2 mutations 87%*	*Short stature* 45% *Malnutrition* 38% *Bone diseases* 37% *Obesity* 9%	*Epilepsy disorders* (81%)
**Knight et al., 2012 (** 32) **Australia**	*Longitudinal population-based data* From the Australian population-based Rett Syndrome Database.	213	Born from 1976	213 *female*, 0 *male* N/A	*MeCP2 mutations 100%*	*Malnutrition* 21.5% *Delayed menarche* 9% *Precociuos puberty 6%* *Obesity* 4.2%	*N/A*
**Baş et al.,2013 (** 33) **Turkey**	*Case report*	1	6 years	1 female Turkish	*MECP2: C455G P152R*, missense mutation	*Precocious puberty*	*Epilepsy disorders*
**Killian et al., 2013 (** 19) **USA**	*Retrospective observational* Through the multicenter RTT Natural History study (RNHS)	802	Born from 1943 to 2010	802 *female*, 0 *male* *American Indian* 0.7% *Asian* 4.4% *Native Hawaiian* 0.3% *Black* 4.2% *White* 86.9% *Mixed black and white* 1.6% *Missing demographic data* 1.9%	*MeCP2 mutations 100%*	*Delayed menarche* 19% *Premature menarche* 13% *Precociuos puberty 10%*	*N/A*
**Bernstein et al., 2019 (** 34) **Germany**	*Case series*	2	11 and 34 years	2 *female*, 0 *male* *Caucasian*	*A* novel *MeCP2 a* variant*: c.1162_1172del; p.Pro388* in both patients	*Precocious puberty*	*Epilepsy disorders* (100%)
**Yang et al., 2021 (** 35) **China**	*Case report and literature review*	1	8 years	1 *female*, 0 *male* *Chinese*	*De novo MeCP2 variant c.1157_1197del*	*Precocious puberty*	*Epilepsy disorders*
**Hamilton et al., 2012 (** 36) **USA**	*Anonymous web-based survey.*	21	10-25 years (average 17.1)	21 *female*, 0 *male* *Caucasian 86%*	*N/A*	*Premenstrual Syndrome* 71% *Dysmenorrhea 76%*	*N/A*
**Humphrey et al., 2020 (** 37) **USA**	*Retrospective cross-sectional chart review and prospective survey.*	77	12-55 years	77 *female*, 0 *male* *Black or African American* 9% *Caucasian* 88% *Other, not specified* 3%	*MeCP2 64.9%* *Unknown* (not tested/results unavailable/unknown pathogenicity) 35.1%	*Dysmenorrhea 61%* *Catamenial seizure 22.1%*	*Epilepsy disorders* 61%
**Motil et al., 2015 (** 38) **USA**	*Cross-sectional, prospective*	50	15.5 ± 9.7 y (2-38 y)	50 *female*, 0 *male* *Caucasian* 64 African American 16% Hispanic 14% Asian 6%	*MeCP2 90%*	*Low bone mineral content* 59% *Low bone mineral density* 45% (decreased bone formation rather than increased bone resorption) Osteocalcin concentrations for all age groups were significantly lower, whereas the concentrations of bone alkaline phosphatase J were significantly higher in the RTT cohort than their respective values	*Epilepsy disorders* 66%
**Jefferson et al., 2014 (** 39) **Australia**	*Clinical trial* From the Australian Rett Syndrome Database (ARSD)	97	Mean age 15 yo (4–30.5 years)	97 *female*, 0 *male* N/A	*MeCP2 90%*	*Fractures* 31.7% *Low mineral density:* - *Lombar* 41.3% - *Total* 44.6% - *Femoral* 78%	*Epilepsy disorders* 75.3%
**Budden et al., 2003 (** 40) **USA**	*Clinical trial* From the Australian Rett Syndrome Database (ARSD)	5	Mean age 12.05 yo (4–30.5 years)	5 *female*, 0 *male*	*MeCP2 60%* *Unknown 40%*	*Low bone volume accompanied by low bone formation rates of* 100%	*Epilepsy disorders* 20%
**Motil et al., 2011 (** 41) **USA**	*Retrospective review, observational*	284	11.7 ± 8.4	284 *female*, 0 *male* *Caucasian* 69% *Hispanic* 19% *African* *American* 5% *Asian* 5% *Native American* 2%	*MeCP2 99%* *Unknown 1%*	*Low vitamin D* 20% *Malnutrition* 15% *Obesity* 4%	*Epilepsy disorders* 57%
**Shapiro et al., 2010 (** 42) **USA**	*Cross-sectional observational study*	49 + 1	Female: 1.9–17 y (mean age, 7.6 3.8 y) Male: 6 years	49 *female*, 1 *male* N/A	*MeCP2 100%*	*Low bone mineral density* 48.9% *Fractures* 11%	*Epilepsy disorders* 31%
**Caffarelli et al., 2020 (** 43) **Italy**	*Retrospective* survey	232	age range 4–33years; mean age 13.8 ± 8.3yrs	232 *female* Caucasian	*MeCP2 100%*	*Scoliosis 51.6%* *Bone fractures 20.9%*	N/A

Selected following the inclusion features of the studies Characteristics of included studies. N/A, not applicable.

## 4 Discussion

In the last two decades, there has been growing evidence that RTT is not only a neurological disorder but potentially affects several organs and apparatus (44). RTT has a complex and heterogeneous clinical appearance (17). Although neurological conditions are predominant, the disease also affects various non-neurological organs. A possible explanation for this multisystemic involvement may be related to the ubiquitous presence of *MeCP2* in peripheral tissues. Multi-system comorbidities, e.g. gastrointestinal, orthopedic, endocrine, or cardiac issues could be more or less prevalent. Notably, endocrine disorders are more frequent in RTT than in the general population even if systematic data are still limited and not univocal. We selected studies published in the last 24 years, focusing on the prevalence and type of endocrinopathies reported in RTT patients.

### 4.1 Short stature

Short stature is reported as one of the most common endocrinological findings in RTT population. Indeed, growth failure is among the supportive diagnostic criteria of RTT, usually observed as decreased velocity of head growth, height, and weight for age (3). To the best of our knowledge, this finding was first described in 1992 by Thommessen et al., reporting a stature below the -2 standard deviation scores (SDs) for sex and age in RTT girls (45). Growth velocity is usually normal until 15 months of age when deceleration is first noticed. In 1993 Schultz et al. underlined the pattern of growth of RTT mainly characterized by deceleration of head growth, followed by deceleration of weight and height measurements. Considering that the typical motor and behavioral alterations of RTT often appear after the second year of life, this growth pattern can be the first clinical element of suspicious for this syndrome (46). In consideration of this typical growth pattern in RTT Tarquinio et al. in 2012 proposed specific Growth Charts for height, weight and BMI for patients with Rett syndrome that have been approved and used by International Rett Syndrome Foundation to avoid a common clinical and research setting in RTT (27).

Regarding possible endocrine disruption in RTT involving growth, Huppke et al. showed that growth retardation - which involved 91.1% of their RTT cohort doesn’t seem to be caused by Growth Hormone (GH) deficiency. Nevertheless, disturbed hypothalamic control cannot be excluded (26). After about 10 years, even Hara et al. investigated short stature and GH deficiency in RTT. Notably, despite patients with epilepsy and Rett syndrome having a delayed growth spurt, the final height wasn’t statistically different from a control group with only epilepsy. Moreover, circulating levels of GH, IGF-1, and ghrelin were not significantly correlated with height in either group (47).

Of course, in RTT nutritional status could negatively affect growth because of the decline in feeding abilities that may occur during ages. Oddy et al. reported that factors such as enteral nutritional support, mobility, breath-holding, and hyperventilation may strongly affect growth in subjects with RTT. Feeding difficulties could impair adequate nutritional intake, so active nutritional management is needed to ensure RTT patients the best opportunity to reach their growth potential (48).

In this regard, in 2001 Reilly et al. underlined that, even if mechanisms causing growth failure are poorly understood, both nutritional and non-nutritional factors are pivotal (48). In the following years, a lot of studies were published about new mutations in the *MeCP2* gene and their association with phenotype. Tarquinio et al. reported a correlation between the subtype of *MeCP2* mutation with growth pattern, showing that growth failure occurs less frequently in case of late truncation mutations. This finding was probably due to mutations that affected patients with milder phenotypes. In contrast, other types of mutations (pre-C-terminal truncation and R270X), may convey several comorbidities that have an impact on growth in RTT, such as oropharyngeal and gastrointestinal dysfunctions, scoliosis, seizures, and osteopenia (27). Therefore, it seems that mutations are correlated to growth velocity but above all with clinical severity. Indeed, growth failure is more evident in case of mutations that lead to greater clinical severity, such as pre-C-terminal truncation and R270X, rather than that associated with a milder clinic phenotype, such as R306C, R133C, and C-terminal truncation (49). Tarquinio et al. found severe growth failure together with microcephaly in 29.2% of their RTT cohort. Interestingly, Huppke et al. showed that most females with RTT and *MeCP2* mutations had a smaller occipitofrontal circumference, shorter length, and lower weight at birth. Indeed, a role of *MeCP2* protein in intrauterine development could be hypothesized (50). Recently, Wong et al. underlined the correlation between growth deficit, malnutrition, and clinical severity of the disease (age of onset, dystonia, deambulation, hand use, and language impairment), reporting a prevalence of 41.6% for short stature and 37.5% for malnutrition (25). This study was also the first to give importance to ethnicity when comparing RTT individuals’ growth patterns to the RTT-specific chart.

Most recently, Pepe et al. carried out a two-center observational study on a pediatric cohort of 51 RTT pediatric patients with different genetic mutations (*MeCP2, CDKL5, FOXG1*). Short stature was reported as the most common endocrine disorder (47.1%), after excluding secondary causes of growth restriction (celiac disease and GH deficiency). Interestingly, all the patients with short stature exhibited *MeCP2* mutations (23).

### 4.2 Weight disorders (obesity and malnutrition)

Obesity and malnutrition represent the opposite manifestations of an alteration in weight balance in RTT patients. They are both among the most common endocrinopathies reported in RTT, with malnutrition being more prevalent than obesity. *MeCP2* deletion induces dysregulation of lipid metabolism along with significantly increased lipogenic enzyme gene expression and may alter the expression of hypothalamic genes related to feeding regulation. In this direction, an interesting experimental study carried on in 2013 showed that *MeCP2* positively regulates POMC expression in the hypothalamus. The absence of *MeCP2* in POMC neurons leads to increased DNA methylation of the POMC promoter, which, in turn, downregulates POMC expression, leading to obesity in mice with increasing leptin resistance. Therefore, in POMC neurons *MeCP2* seems to be essential in energy homeostasis regulation (51).

Experimental animal models showed that in female heterozygous *MeCP2* -null mice fed with a high-fat diet, a dysregulation of food intake in the hypothalamus and dopamine reward circuitry can be observed, accelerating the development of obesity (52). Another hypothesis correlates genetic *MeCP2* disorders and syndromic manifestations. The first *de novo* mutation of *MeCP2* was described in 2002 in a male patient (53), associated with moderate intellectual disability, hypotonia, obesity, and gynecomastia. Couvert et al. also observed obesity and neurological disabilities in three patients with *MeCP2* mutations (54). Although obesity in these patients could be an occasional finding, it might also suggest a role for *MeCP2* in regulating energy balance. Moreover, some patients with features of Angelman syndrome may also convey mutations in the *MeCP2* gene (55). Nevertheless, the correlation between weight and genotype remains unclear and is mainly investigated in murine studies.

Similarly to short stature, malnutrition is often present in RTT patients in the first years, soon after the onset of neurological regression. Already in 1992, studies on weight and nutrition indicated that underweight was likely to be the consequence of oral-motor dysfunctions and poor self-feeding abilities. These problems, despite preserved appetite, lead to reduced food intake with subsequent growth retardation, with the mean energy intake being 66.9% in RTT according to the US recommendations (56) for age and 107.8% for body weight (45).

Weight deficit in RTT seems to be related to alterations in energy balance (57). In a Polish case-control study of 49 Rett adolescent females, malnutrition was described in 38.78% and obesity in 10.2% of the case group. In comparison to Polish healthy girls, the diet of girls with Rett syndrome was characterized by a significantly lower energy value, and less carbohydrate, protein, fiber, calcium, and iron content. All comorbidities that include feeding disorders requiring crushing, chopping, or blending of food, and depending on the mobility of patients will compromise alimentation and consequently weight status (30). Wong et al. reported that 41.03% of their Taiwanese RTT cohort had inadequate energy intakes, especially in the severe growth deficit group with age ≤ 18 years; nevertheless, there was no significant difference regarding the nutrient intake between severe growth deficit and mild or moderate growth deficit. This suggests that other factors in addition to nutrient intake may play a role in the growth pattern of those with severe growth deficit (25). As mentioned above, Wong et al. underlined that nutritional status and dietary intake in RTT may differ among various ethnic groups and countries.

Conversely, several studies reported a normal BMI in RTT patients. This is probably due to concomitant weight and height deficiency that typically affects RTT (27, 31).

Moreover, it should always be kept in mind that *MeCP2* is a ubiquitous protein, expressed even in the gastrointestinal (GI) tract. Alterations in the digestive system could be present and contribute to poor alimentation and underweight. In a cohort of 983 females with RTT, 92% had GI problems (gastroesophageal reflux, constipation, straining with bowel movements, and passage of hard stools) and a small percentage (4.4%) also have biliary tract disease, which may have a fatal outcome (31). In RTT, disruption of swallowing may occur at any stage causing significant alteration of the eating and drinking phases and resulting in dysphagia (disorders that may occur in the oral, pharyngeal, or oesophageal stage). Still in 2001, when RTT’s genetic alterations were almost unknown, Reilly et al. described malnutrition in 26% of 59 Australian RTT girls and concluded that the consequences of dysphagia for both females with Rett syndrome and their caregivers are affecting health, development, and general well-being (28).

While several studies have been published regarding malnutrition in Rett population, little is known about the impact of obesity. According to Motil et al., 9% of female individuals with RTT were diagnosed as overweight or obese (31). Although RTT has been associated with obesity, the underlying mechanism has not been elucidated yet. The prevalence reported is quite similar in different studies, but of course, also ethnicity, social condition, and food availability should be considered as influencing factors. For example, in a transversal Brazilian study by Schwartzman et al., malnutrition affected 37% of RTT females, and obesity 7.4% (29). In a recent pediatric observational Italian study, the prevalence of malnutrition and obesity were respectively 25.5% and 19.6% (23).

Few studies demonstrated weight-for-height excess, as well as overweight among children with autism spectrum disorder, hypothesizing an eating disorder under neurological disorders. Research has shown that children with autism may be more likely to have weight problems, such as being overweight. This could be due to underlying eating disorders associated with neurological conditions (58).

The role of leptin, a peptide hormone mainly produced by white adipose tissue, has been investigated in the regulation of body weight and energy expenditure. Blardi et al. reported higher leptin levels in RTT patients than controls positively correlated with age and BMI (59). Unexpectedly, the increased leptin concentrations were not always associated with obesity. Authors hypothesized that in patients with RTT, leptin levels might be related to factors other than weight balance, such as the regulation of neuroendocrine and immune functions, and infections, frequently present in RTT due to respiratory alterations (55).

The assessment of the nutritional status of the individual with RTT serves as a guide to nutritional intervention such as supplemental formula use, feeding gastrostomy, etc. Regular monitoring of growth parameters, including weight, height, and BMI, is necessary. However, measurements in RTT are sometimes challenging since some patients are unable to stand on their own or have spinal deformities, while the mere comparison of body weight cannot be concluded without reference to body height and height gain.

### 4.3 Gonadal function

#### 4.3.1 Precocious puberty

Abnormalities in pubertal onset represent a prevalent aspect of endocrine comorbidity in RTT. The most common alteration of puberty in RTT syndrome is precocious puberty (PP). Data about pubertal disorders come mainly from case report series. Interestingly, this finding has been reported more recently than short stature, malnutrition, and bone dysfunctions. Mainly, the relationship between puberty and RTT was evaluated in murine experimental trials. In 2009, Garcia-Rudaz et al. showed that in a mouse model of RTT, the expression of FXYD1, a modulator of Na(+)K(+)-ATPase activity, was increased, thus favoring puberty onset by maintaining GnRH neuronal excitability (60). Evidence from another mouse model of RTT suggested that regulation of gonadotropin-releasing hormone by *MeCP2* could influence the onset of puberty. For example, in case of *MeCP2* dysfunction (truncating deletion), altered estrogen receptor expression could provide the earlier stimulation of breast development (61).

A recent study by Yang et al. underlined the coexistence of PP and more severe neurological disorders (abnormal EEGs and intractable epilepsy) (35). In this regard, previous studies have shown that epileptic activity, especially mediated through the amygdala, alters reproductive function, including changing ovarian cyclicity in females and altering sex steroid hormone levels in both sexes (62). Different studies demonstrated that *MeCP2* co-3 localizes with GnRH within GnRH neurons in the hypothalamus (63, 64). It seems that *MeCP2* could be a potential player in the regulation of human pubertal timing. In this regard, some authors reported rare heterozygous *MeCP2* mutations in girls with central precious puberty (CPP), with or without neurodevelopmental abnormalities (63–65).

In 2013, Bas et al. reported for the first time a case of central PP in a 6-year-old RTT Turkish girl with *MeCP2* missense mutation (C455G P152R) (33). In 2015 Knight et al. investigated pubertal development in a longitudinal population-based study in RTT. They included 213 female patients born since 1976, using the Australian Rett syndrome database, and reported 6% of cases of precocious puberty, despite delayed menarche in 9%, indicating that the pubertal timing in RTT may be abnormal with anticipated onset but longer duration. These features may be genotype-dependent and could be influenced by malnutrition, growth issues, and bone maturation (32). Likewise, Killian et al. examined individuals with both clinical diagnoses of Rett syndrome or mutations in *MeCP2* using the US Natural History Study database (19). More than 25% of them initiated puberty early yet entered menarche late; only 4% experienced delayed thelarche. This strange trajectory in RTT could be again related to the effect of BMI and genetics. But while BMI was associated with the age of onset of thelarche and pubarche, mutation type was significantly associated with menarche, with the most severe mutation (for example R168X and R255X) predicting later menarche. However, these findings raised additional questions regarding pubertal trajectory in RTT.

Even if previous studies on gonadal and adrenal steroids suggested normal sex hormones in RTT (26), recently there has been growing clinical evidence about the association between *MeCP2* mutations and precocious puberty. In 2019, Bernstein et al. described 2 cases of RTT girls with *MeCP2* deletion together with intellectual disability, obesity, metabolic syndrome, macrocephaly, and precocious puberty. The genetic analysis showed in both a new variant, c.1162_1172del; p.Pro388 not listed in the Rett database (34). Both patients did not show the full manifestations of Rett syndrome symptoms but presented with intellectual disability and seizures. Moreover, both displayed precocious puberty and obesity.

Recently a multiethnic cohort of 404 patients with idiopathic CPP was tested for genetic study including the research for *MeCP2* mutations by Sanger sequencing (63). It was found that four rare heterozygous *MeCP2* variants (*de novo* missense and insertion), were present in seven girls with CPP. These four *MeCP2* variants identified in CPP girls have not been associated with the Rett syndrome phenotype, except for one case (p.Arg97Cys variant). The other patients presented with different clinic phenotypes, including obesity and autism or microcephaly.

Again, Pepe et. al, in their observational RTT cohort study reported a prevalence of PP of about 15.4%, mainly due to *MeCP2* mutations, higher than other prevalence studies. Of course, the interpretation of this finding should also consider ethnic diversity, in addition to the Western trend to PP recorded in the last decades among healthy girls (23).

#### 4.3.2 Menstrual irregularities

Recent studies revealed that the average age of menarche in females with Rett syndrome was 12.2 years (SDs +/- 5.4 years). However, data are scarce and not univocal. Killian et al. found that 13% of RTT girls reached menarche prematurely compared to the general population, whereas 19% experienced delayed menarche. Menarche occurred earlier in those with a milder mutation genotype (19). Developmental disability often makes menstrual discomfort challenging for caregivers (66), and patients cannot adequately communicate their symptoms and needs. Few studies evaluated specifically this aspect in RTT girls. Hamilton et al. used an anonymous web-based survey for girls 10-25 years old recruited from Rett syndrome LISTSERV in 2009 (36). The results showed that the mean age of menarche was 11.7 years (SD +/- 2.0 years), that the majority of the girls (62%) reported periods of 3-7 days, and 48% of them were using hormonal contraception at the time of the survey; at least one symptom of dysmenorrhea was reported in 76% of cases, with cramps and low back pain the most frequently presented symptoms.

An interesting retrospective cross-sectional chart review and prospective survey in 2020 described data on features of menstruation and menstrual-related symptoms in a large cohort of RTT girls, reporting the prevalence, types, and efficacy of hormonal treatment (37). The most frequent symptoms include dysmenorrhea (61.0%) and emotional lability (49.4%). Features of menstruation in RTT were like those in the general population, except for an increase in catamenial seizure.

Menstrual cycle irregularities were a common finding in the most recent Italian RTT cohort study, accounting for about half of all the endocrinopathies reported in the study population (23), predominantly oligomenorrhea and secondary amenorrhea. Remarkably, the entire group of patients suffered from body weight alterations, both overweight and underweight. Furthermore, premature ovarian failure (POF) was diagnosed in two RTT patients with secondary amenorrhea, enhancing a possible link between RTT and POF. Such association is still almost unknown, even if *FMR1* (Fragile X messenger ribonucleoprotein 1) and *FMR2* genes, often involved in POF with genetic etiology, are located on the long arm of chromosome X (Xq27.3 325 and Xq28, respectively), next to *MeCP2*, and therefore mutations of these genes might explain POF in RTT (67).

### 4.4 Thyroid disorders

In consideration of documented *MeCP2’s* function in maintaining the mature neuronal state, its absence and alterations could have several implications for the balance between synaptic excitation and inhibition (68). The histological findings observed in *MeCP2*-deficient mice especially the loss of parvalbumin neurons in the cerebral cortex share many similarities with that of hypothyroid mice. A typical neuropathological finding caused by thyroid hormone insufficiency is a decrease in the parvalbumin of GABAergic neurons (69). Parvalbumin is a protein expressed in GABAergic neurons in the central nervous system. immature cortical formation in the cortex of *MeCP2* -deficient mice, a phenomenon also observed in hypothyroid mice (70).

In a recent experimental study, human-induced pluripotent stem cells (iPSC) were used to generate *MeCP2* knockout neuronal progenitor cells and adult neurons and then investigate the expression of genes associated with thyroid hormone homeostasis (deiodinases and transporters). *MeCP2* -knockout cells cause alterations in thyroid hormone-related genes, such as hormone transporters and deiodinases (71). In consideration of these results, one hypothesis is that *MeCP2* probably has a significant influence on the assembly of the thyroid system in the body.

Literature data about thyroid disorders in RTT patients are extremely scarce and contrasting. Cooke et al. reported for the first time in 1995 that patients with RTT might have thyroid dysfunction, such as a significant decrease in serum total FT4 concentration. Their study found minimal changes in the TSH levels in RTT patients with no evidence of clinical hypothyroidism. Some of the subjects included with RTT were treated with anticonvulsant drugs. In conclusion, lower thyroid function in RTT was related to the thyroid gland’s primary dysfunction and altered hypothalamic-pituitary axis function, considering that even T4-binding proteins were normal (72). Another study by Huppke et al. showed normal age-appropriate plasma values for FT4, TSH, and TSH-night rhythm (26).

Recently, two Italian studies focused on the prevalence of thyroid disorders in RTT. The first one by Stagi et al. (24) reported that FT3 and TSH levels were higher in RTT patients versus controls, even if without reaching statistical significance; moreover, FT4 levels were significantly higher in the RTT group (17.7%), especially in those with *CDKL5* deletions. This finding may support the hypothesis that in many patients with RTT, higher FT4 levels with normal TSH could reflect the attempt to increase and maintain the action of TSH at the central nervous system level, and *MeCP2* is thought to be necessary to stabilize the mature neuronal state (23, 68). Instead, few data are available to understand the molecular connection between *CDKL5* deletions and thyroid dysfunction. The second Italian study by Pepe et al. (23) reported thyroid disorders in approximately 10% of 51 RTT patients, ranging from autoimmune thyroiditis to central hypothyroidism and hyperthyreotropinemia, with *MeCP2* mutations being the most frequent genotype. Although there is not a clear explanation regarding the behavior of *MeCP2* transcripts and translated products which differs between organs, it seems that thyroid hormones may have a pivotal role in the expression of *MeCP2* and the relationship between thyroid function and *MeCP2* might be that of reciprocal interactions rather than one-way interactions (73).

### 4.5 Bone Health and Orthopedic Issues

Evidence from both human and animal studies supported the hypothesis that *MeCP2* mutations could be associated with altered epigenetic regulation of bone-related factors and signaling pathways, including RANKL/RANK/OPG system. Nevertheless, further studies are needed to better understand the role of *MeCP2* in bone homeostasis (74).

Bone health concerns are relevant in RTT due to motor impairment and skeletal abnormalities. Indeed, it was among the first endocrinopathies described in RTT patients (75, 76). Female patients with RTT often suffer from altered bone health with decreased mineral content, decreased mineral density, and an increased fracture rate three to four times that of normal females. Common findings are orthopedic problems including scoliosis and joint contractures (76). Scoliosis is related to the lack of walking action, whereas it seems to be unrelated to the loss of hand skills or hand stereotypes. Several markers, such as osteocalcin or bone-specific alkaline phosphatase, were found to be reduced in RTT, thus enhancing low bone turnover in these patients (77). Such alteration of bone mineral deposition may be caused by vitamin D deficiency typical of RTT population, together with the lack of spontaneous mobilization (41).

Budden et al. studied osteopenia and osteoporosis in RTT girls analyzing bone remodeling by quantitative bone histomorphometry. They found a slow rate of bone formation, thus negatively influencing development and accumulation of peak bone mass and contributing to decreased bone volume in Rett syndrome (40). In the same direction, Motil ed al. underlined that decreased bone formation, rather than increased bone resorption, may partially explain the deficits in bone mineral mass in RTT (low bone mineral content 59%, low bone mineral density 45%*)* and that dietary factors, but not hormonal or inflammatory markers, were associated with altered bone mineral status. This requires adequate calcium, protein, and phosphate diet assumptions, to improve bone health in RTT. Osteocalcin concentrations for all age groups were significantly lower in RTT study population, whereas the concentrations of bone alkaline phosphatase were significantly higher (38).

Even if the normal ghrelin/GH/IGF-1 axis stimulates longitudinal bone growth, Caffarelli et al. reported that plasma levels of ghrelin did not reflect longitudinal bone growth in female RTT patients within a growing period (78).

Jefferson et al. used densitometry (DXA) to evaluate bone density and content, analyzing how factors such as genotype, epilepsy, BMI, and mobility might affect these parameters. They confirmed low bone mineral density and bone mineral content in RTT, particularly at the femoral neck DXA. This finding seemed to be directly associated with the type of *MeCP2* mutation, especially p.R168X and p.T158M (39). Indeed, the lack of *MeCP2* may reduce bone density through osteoblastic dysfunction (79).

Shapiro et al. reported bone mineral density values 2 SD below age-related norms in 48.9% of RTT patients (42). Motil et al. observed low bone mineral content or fractures in 37% of RTT patients, with older RTT females showing more severe phenotypes rather than younger ones (31).

Finally, the prevalence of osteopenia and osteoporosis reported in the observational study by Pepe et al. is lower than the previous study (19.6%), probably because of infrequent testing of bone status outside the research setting or particular clinical situations (23).

The last clinical guideline for the management of bone health issues in RTT highlighted the need for both fracture and low bone densitometry for a diagnosis of osteoporosis in RTT. Increasing physical activity and initiating calcium and vitamin D supplementation is recommended in Rett patients (74, 80).

## 5 Limitations of the study

The main limitation of this review is the heterogeneity of outcomes and design of the studies included. The majority of them were not focused on the primary aim of the endocrinopathies in RTT. The Rett population included in this review was not homogenous in terms of sex, age, ethnicity, country, and genotype. However, this feature could be seen as a point of value of the review, as it collected multifaceted aspects of the same genetic syndrome. The number of patients included was often small, except for studies based on registers. Only a few studies recruited male RTT population and genotype other than *MeCP2* mutations.

Nevertheless, a common aspect of the studies selected was the attention to the type of mutation in *MeCP2*, the prevalence of endocrinopathies, and their severity, in addition to the focus on genotype-phenotype correlation especially in the severe Rett forms and mutations. Of course, systematic data are needed to confirm these findings.

Finally, the features of associated epileptic activity in RTT (type and severity, age of onset, control of disease, drugs), were not constantly reported in the studies available, even if it is known to potentially influence endocrine function.

Despite some limitations, to the best of our knowledge, this is the first systematic review specifically focused on reporting the prevalence of endocrinopathies in RTT. The results showed that endocrine disorders represent a common finding in RTT, and therefore should be adequately investigated, to improve the quality of life and the care of these patients. Due to the lack of specific recommendations, we hope that our effort could highlight the need of periodic endocrinological follow-up, to prevent and detect endocrinological comorbidities at an early stage. A proposal for RTT patient’s endocrinological management is synthesized in **Table 2**.

**Table 2:** Proposal for endocrinological screening and follow-up in Rett syndrome, from diagnosis to adulthood.

ENDOCRINOLOGICAL MANAGEMENT	INFANCY AND CHILDHOOD	ADOLESCENCE	ADULTHOOD
*Growth evaluation*			
*Linear growth*	√	√	NA
*Weight gain and BMI*	√	√	√
*Tanner stage*	√	√	NA
*IGF-1 and GH secretion*	*in case of short stature and/or growth deceleration	*in case of short stature and/or growth deceleration	NA
*Bone age*	√	*in case of short stature and/or growth deceleration	NA
*Thyroid function*			
*FT4, TSH*	√	√	√
*TPO-AB, TG-AB*	√	√	√
*Thyroid ultrasound*	√	√	*in case of thyroid disorders, goitre or family history
*Metabolic assessment*			
*Blood pressure*	√	√	√
*Waist circumference*	*if BMI > 2.0 SDS	*if BMI > 2.0 SDS	*if BMI > 2.0 SDS
*Blood glucose*	√	√	√
*Insulin, OGTT, Hba1c*	*if BMI > 2.0 SDS	*if BMI > 2.0 SDS	*if BMI > 2.0 SDS
*Lipid profile*	√	√	√
*Gonadal function*			
*LH, FSH, PG, E2*	*in case of clinical signs of precocious puberty	*in case of delayed puberty or menstrual irregularities	*in case of menstrual irregularities
*LHRH test*	*in case of clinical signs of precocious puberty	*in case of pubertal alterations	NA
*AMH*	NA	*in case of amenorrhea	*in case of amenorrhea
*FSH, LH, T, AMH, INIBIN B*	NA	*in case of male disorders of pubertal development	*in case of male disorders of pubertal development
*Pelvic ultrasound*	*in case of clinical signs of precocious puberty	*in case of delayed puberty or menstrual irregularities	*in case of menstrual irregularities
*Bone health*			
*Calcium-phosphorus metabolism*	√	√	√
*DEXA*	*if necessary	Every 2 years	Every 2 years
*25-OH vitamin D supplementation*	√	√	√
*Other*			
*Nutritional counselling/support*	√	√	√

*NA, Not applicable.

BMI, body mass index; SDS, standard deviation score; DEXA, dual-energy X-ray absorptiometry.

√, to be performed at diagnosis and periodically thereafter.

## 6 Conclusion

Rett syndrome is a severe neurological disorder that has increasingly emerged in recent years. Multiple organs and apparatus can be involved, with a broad spectrum of manifestations. The present systematic review shows that endocrinopathies are not rare in RTT patients, with malnutrition, short stature, pubertal abnormalities, and bone disorders being the most frequent endocrinological findings. Interestingly, patients with *MeCP2* alterations seem at higher risk for developing endocrinopathies because of this protein’s ubiquitous distribution. These data highlight the need to recommend a specific endocrinological evaluation and follow-up in all RTT patients, to ensure a better quality of life in a multidisciplinary approach.

## Data Availability

The raw data supporting the conclusions of this article will be made available by the authors, without undue reservation.
